# Long-term climate change in the D-region

**DOI:** 10.1038/s41598-017-16891-4

**Published:** 2017-11-30

**Authors:** Mark A. Clilverd, Roger Duthie, Craig J. Rodger, Rachael L. Hardman, Keith H. Yearby

**Affiliations:** 1 British Antarctic Survey (NERC), Cambridge, UK; 2 Department of Physics, University of Otago, Dunedin, New Zealand; 3 Department of Control Engineering, University of Sheffield, Sheffield, UK; 4 Present Address: School of Mathematics and Statistics, University of Sheffield, Sheffield, UK

## Abstract

Controversy exists over the potential effects of long-term increases in greenhouse gas concentrations on the ionospheric D-region at 60–90 km altitudes. Techniques involving *in-situ* rocket measurements, remote optical observations, and radio wave reflection experiments have produced conflicting results. This study reports a novel technique that analyses long-distance subionospheric very low frequency radiowave observations of the NAA 24.0 kHz transmitter, Cutler, Maine, made from Halley Station, Antarctica, over the period 1971–2016. The analysis is insensitive to any changes in the output power of the transmitter, compensates for the use of different data logging equipment, and can confirm the accuracy of the timing systems operated over the 45 year long record. A ~10% reduction in the scale size of the transmitter nighttime interference fringe pattern has been determined, taking into account the quasi-11 year solar cycle. Subionospheric radiowave propagation modeling suggests that the contraction of the interference fringe pattern about the mid-latitude NAA transmitter is due to a 3 km reduction in the effective height of the nighttime ionospheric D-region over the last 45 years. This is consistent with the effect of enhanced infra-red cooling by increasing greenhouse gases.

## Introduction

The long term increase in greenhouse gas concentrations in the atmosphere during the 20^th^ and 21^st^ centuries are expected to drive an increase in temperature in the troposphere, i.e., 0–~15 km altitude^1^. In the lower, middle and upper stratosphere (~15–50 km) cooling has been observed in satellite, radiosonde, and lidar measurements^2^. Rocketsonde measurements undertaken at tropical and sub-tropical locations have confirmed the cooling trends for 35–50 km, and provided additional observations of long-term cooling trends up to 63 km^3^. At these altitudes long-term changes of stratospheric ozone concentration can influence the cooling trends observed by up to a third^4,5^ particularly at high polar latitudes associated with the ozone hole^2^. At altitudes above ~100 km a decrease in temperature is expected through the mechanism of infra-red cooling^1^. As the thermosphere cools it contracts, with the resultant effect that satellite and space debris orbital lifetimes increase^6^. The ionosphere well above ~100 km also exhibits a decrease in height as the scale height becomes smaller^7^, consequently features such as the ionospheric F2-layer peak height have been experimentally observed to reduce over time^8^.

In the mesospheric altitude region, located between the thermosphere and the stratosphere, i.e., 50–100 km, long term trends are less clear and more uncertain^9^. A comprehensive synthesis and evaluation of mesospheric long-term temperature trends^10^ confirmed the occurrence of cooling in the lower mesosphere (~50–70 km), while identifying the mesopause (~80–100 km) as an altitude region which exhibits no significant trend, amid large uncertainties. There are three main methods of observing long-term trends in this region: *in-situ* rocket measurements, remote optical observations, and radio wave reflection experiments. Rocket measurements of mesospheric temperatures between ~50–85 km in the polar summer, using falling spheres, suggest a “nearly zero temperature trend”^11^. Optical observations of mesospheric layers initially suggested a decrease in layer height over time e.g., refs^12,13^. However, by extending the sodium lidar dataset, and taking into account seasonal variations in the height of the sodium layer, updated analysis indicates no long-term change in the vertical distribution of atmospheric sodium between 65–110 km, and no clear mesospheric temperature trend^14^.

A model simulation of the period 1950–2003 using the Whole Atmosphere Community Climate Model (WACCM) showed no significant long-term temperature trend near the mesopause (~80–90 km), although cooling trends were determined in the stratosphere below, and in the thermosphere above^15^. A similar result was found using the Hamburg Model of the Neutral and Ionized Atmosphere (HAMMONIA) in a CO_2_ doubling experiment^16^. Using 20-year simulations with a doubling of CO_2_ above the then current concentrations, cooling was found everywhere above the tropopause, but with the smallest effect occurring at the mesopause.

In contrast, there has been an observed decrease in low frequency (LF) radio wave reflection heights between 80–90 km[e.g.^17^]. Recent analysis of phase-height measurements of LF waves (162–164 kHz) made at ~50 °N show a decrease in height of 114 m per decade from 1959–2009 at 82 km^18^. The measurements were made for constant solar zenith angles of 78.4°, i.e., during daytime conditions where the ionosphere is being strongly driven by solar EUV characteristics^19^. The LF long-term decreasing height trend over Europe was attributed to the CO_2_ greenhouse effect, although the influence of the quasi-11 year solar cycle in ultraviolet radiation and shorter timescale oscillations (i.e., the El Nino Southern Oscillation, ENSO, and the quasi-biennial oscillation, QBO) were also apparent in the dataset^18^.

One technique suggested for monitoring the lowest altitudes of the D-region, but not undertaken until now, is the analysis of very low frequency (VLF) radio wave propagation over very long distances^9^. The advantage of the VLF technique over many of the others comes about because of the long distance integration of the measurement compared to relatively small-scale coverage by rocket, optical cameras, and higher frequency radio wave observations. A technique has been identified^20^ of investigating the characteristics of the nighttime D-region using the timing of abrupt decreases in signal amplitude (‘fading’) of man-made transmitter signals caused by mode conversion^21^ associated with the passage of the sunrise terminator^22^. The timing of each amplitude fade is related to the specific location in the upper altitudes of the Earth-ionosphere waveguide (60–80 km) where destructive interference takes place, producing a modal minima^23^. The positions of these modal minima locations are determined by the underlying nighttime electron density profile characteristics of the D-region, typically for reflecting electron number densities of <300 el/cm^3^ (75–85 km at night^24^). The advantage of investigating long-term trends in the nighttime D-region comes from the lack of the direct influence of solar EUV on the behavior of the electron density profile, thereby increasing the sensitivity of the analysis to any anthropogenic changes. In this study we develop the amplitude ‘fading’ technique, and use it to compensate for many of the instrumental/experimental factors that can make long-term comparisons so problematic.

**Figure 1:**
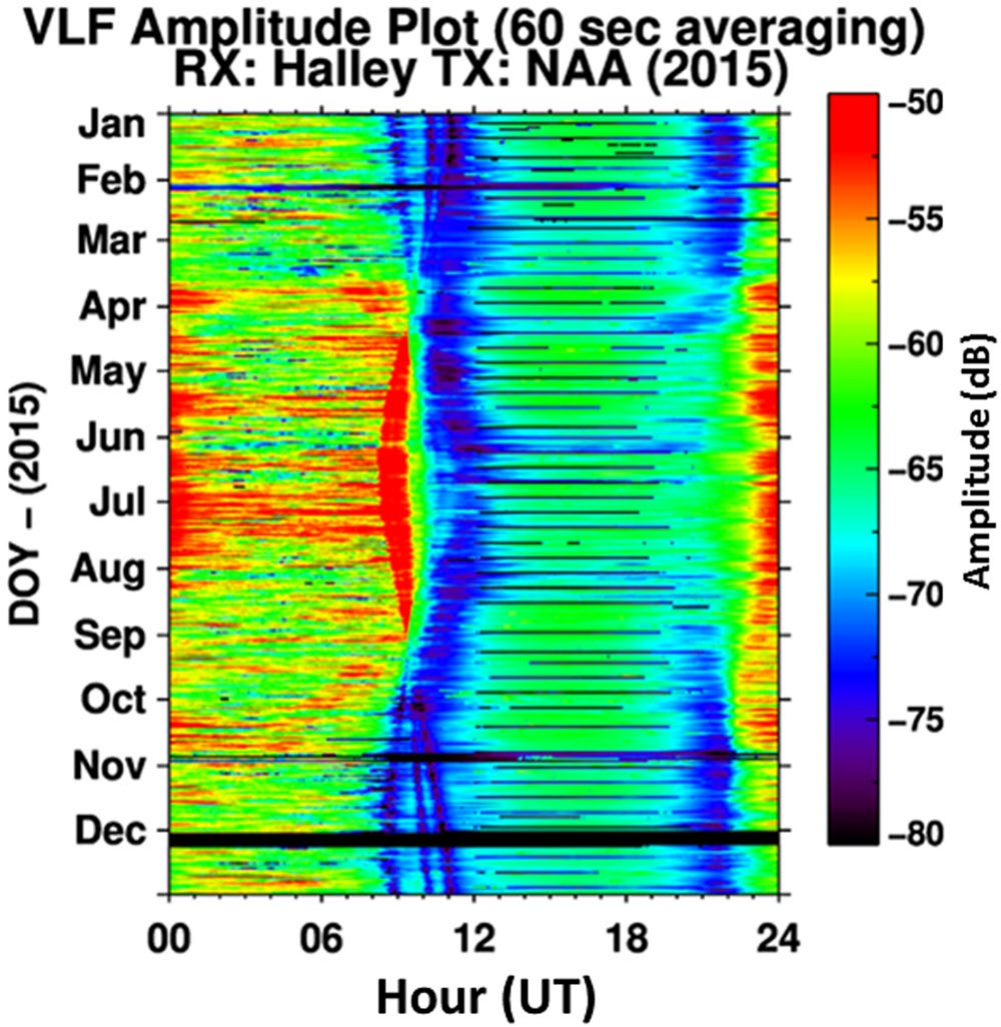
The amplitude of NAA received at Halley in 2015.Amplitude fading due to the passage of the sunrise/sunset terminator along the transmitter-receiver great circle path can be seen as features with blue/black colouring. During November three periods of decreased amplitude can be seen during early morning at 08–11 UT, changing in time as sunrise times change seasonally. Black horizontal stripes indicate transmitter off-times (typically 12–20 UT).

Figure 1 shows an example of the seasonal variation in the amplitude of the NAA transmitter (Cutler, Maine, USA) observed by a VLF receiver system located at Halley Station, Antarctica. The plot shows 60 s averaged amplitude values for 2015 with fading periods easily identified by repeatable, daily, low amplitude features, represented by blue on the colour scale. Multiple sunrise fades can be seen to occur in November-December at ~08–11 UT. The fades are caused by the presence of modal minima at several different locations, with seasonal variations caused by the change of the time of sunrise at a given (known) location rather than a change in the location of the modal minima^20^. However, any long-term change in the characteristics of the electron density profiles of the D-region will change the location of the modal minima, and cause the time of the amplitude fade to change [e.g.^25^]. Changing times of signal fading outside of those expected from seasonal sunrise variations then acts as an absolute litmus test for changing ionospheric conditions^26^. The precise timing of the amplitude fades in November-December over the last 45 years is the focus of this study.

### Determining the time of sunrise amplitude fades

The long-term study of the timing of man-made transmitter signals is potentially made complicated by any changes in transmitter location, frequency, power output, receiver location, logger type, amplitude calibration, and receiver timing quality. Fortunately, several of these factors are constrained by the experimental technique used in this study. Data are analysed from a VLF magnetic loop antenna system located at Halley Station, Antarctica (75°36′S, 26°12′W) that has been in operation since 1967, when it was installed to support the Ariel 3 and 4 satellite missions^27^. Since then, the amplitude of the NAA VLF transmitter (24.0 kHz, Cutler, Maine, 44°39′N, 67°17′W) has been logged intermittently using a series of five different instruments (see Methods section for a more detailed description).

The NAA transmitter has been transmitting with 1 MW output power since 1961, and has changed frequency only once, from 17.8 kHz to 24.0 kHz in 1983. Receiver system setup, and receiver amplitude calibration uncertainties are constrained in this study by only having to identify the time at which amplitude fades occur – the main requirement being that the signal remains above the local noise floor during the measurement. There is no requirement for the receiver system to remain well calibrated over the 45 years of this study, nor is there a requirement for the long-term output power of the transmitter to remain constant. The change in transmitted frequency from 17.8 kHz to 24.0 kHz in 1983 did result in a change in the location of modal minima features generated by the transmitter. However, the dimensions of the interference fringe pattern that surrounds the transmitter^28^ are determined by the frequency used, and therefore in this study, modal minima locations and their equivalent distances from the transmitter can be expressed in terms of the equivalent 24.0 kHz fringe pattern.

**Figure 2:**
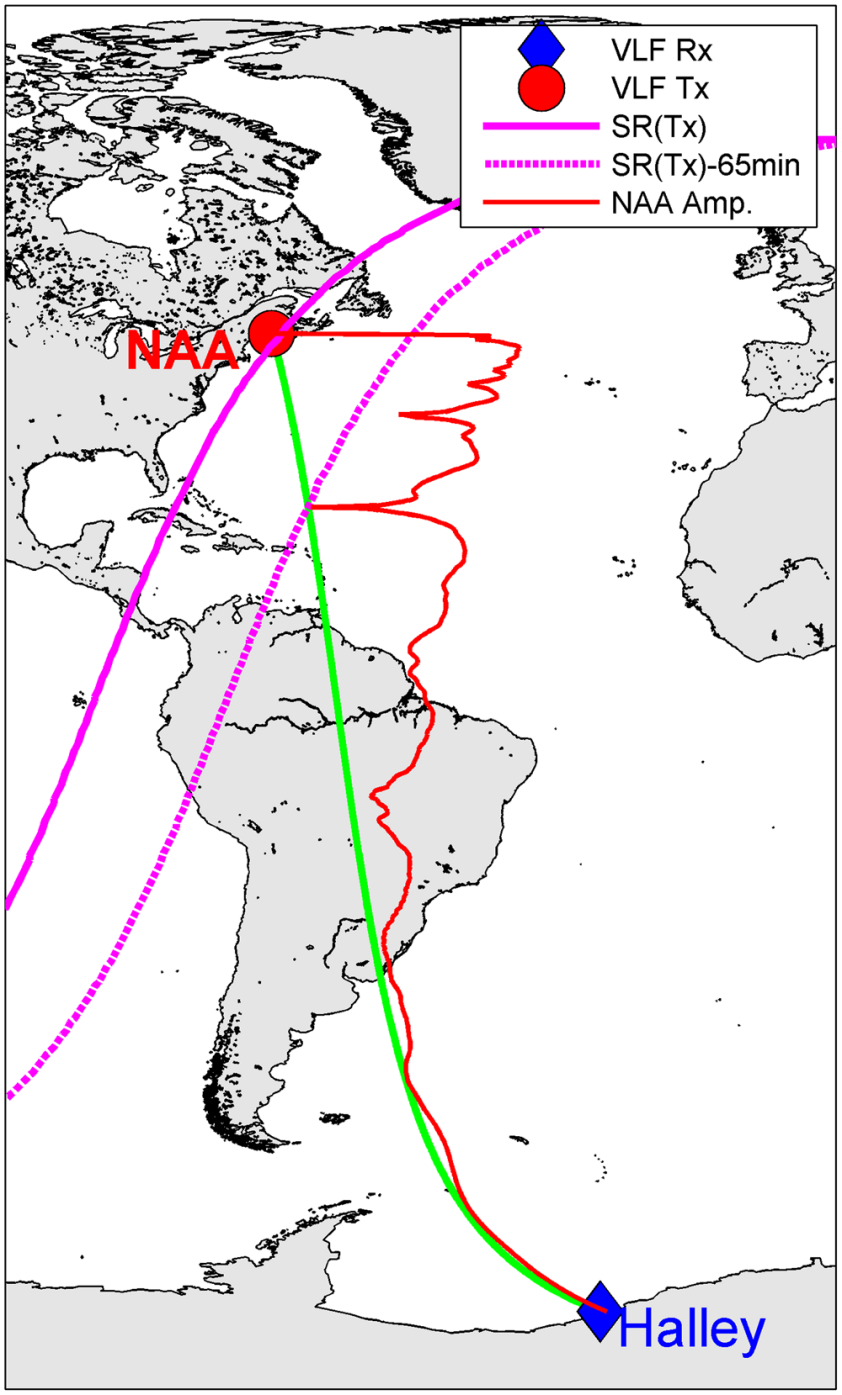
A map of the great circle path from the NAA transmitter (red circle) to Halley Station in Antarctica (blue diamond). A representative nighttime amplitude variation along the path of NAA is shown as a red line. Low amplitude levels occur when the line approaches the great circle path. Sunrise terminator times for 28 November are shown by magenta lines, indicating when sunrise occurs at the transmitter, and at a modal interference minima located ~2000 km from the transmitter. Map generated using Matlab (ver R2016b, https://www.mathworks.com/products/matlab.html).

Figure 2 shows the great circle path (GCP) of the subionospheric radiowaves transmitted from NAA to Halley Station (green line). The amplitude of the transmitter signal at 70 km altitude as a function of distance along the GCP is shown in red, with increased longitudinal distance from the GCP representing increased amplitude. The amplitude was calculated using the Long Wave Propagation Code^29^ (LWPC) with D-region electron density profile characteristics specified for nighttime^24^. Deep modal minima (which generate fading features when they interact with the terminator) can be seen as places where the amplitude line suddenly approaches the GCP. Notable locations where this happens are at the transmitter, and in the Caribbean. The location of the minima in the Caribbean would change for different transmitter frequencies (i.e., 17.8 kHz or 24.0 kHz). LWPC was used to investigate the fringe position between two frequencies (f_1_, f_2_) and a robust relationship of fringe distance, d_2_ = d_1_(f_2_/f_1_)^1.12^ was found for a range of transmitter frequencies. An example of the position of the sunrise terminator in November as it passes overhead of the NAA transmitter is shown by the solid magenta line, with the position 65 minutes earlier shown by the dashed magenta line. The intersection of the terminator lines with the locations of modal minima on the GCP indicate that sunrise fading should occur with a spacing of ~65 minutes in this example.

Over 45 years, involving five different amplitude-logging systems at Halley^30,31^, the issue of accurate clock timing is critical, particularly for this type of study. Fortunately, the time at which the sunrise terminator generates an amplitude fade as it passes overhead of the transmitter is an ever-present feature in the logged data. In this study, the time of sunrise at NAA as observed in the amplitude data as a deep minimum is used to confirm that the system clocks are set accurately (to within 45 seconds) for each year of analysis undertaken.

### Changes in interference fringe patterns

**Figure 3:**
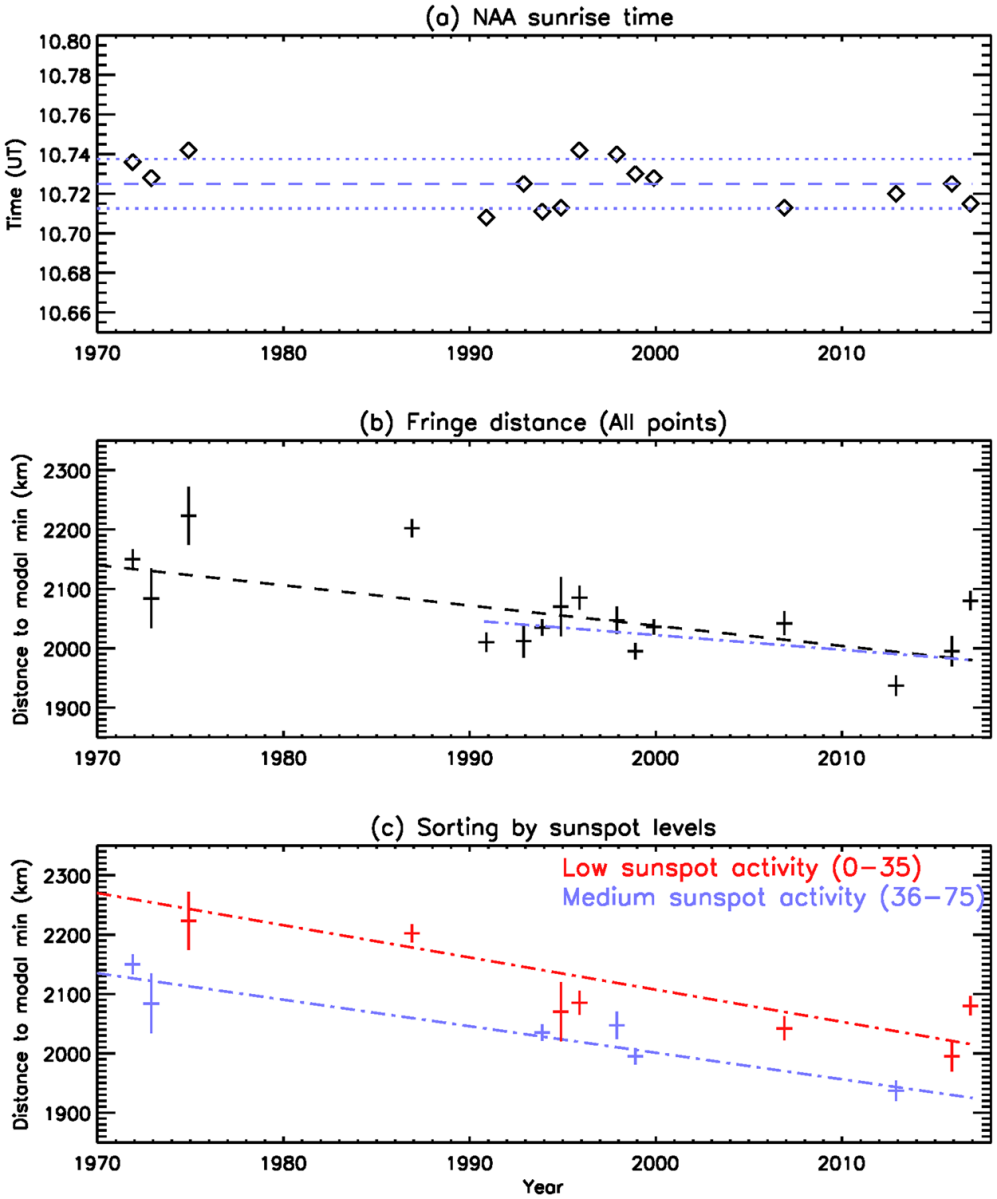
(**a**) The times of sunrise fades overhead of the NAA transmitter during the 45 year study period. The 10:43:30 UT average, and 45 s either side are indicated by the blue dashed and dotted lines respectively. (**b**) The calculated distance of the penultimate sunrise amplitude fade from the NAA transmitter. Normally distributed standard deviation errors bars are shown as vertical lines, and a linear best fit line indicates an interference fringe pattern contraction of 3.6 km/yr over the whole dataset. A fit is also shown for just the 1990–2016 data points (blue, dashed-dot line) indicating a contraction of 2.6 km/yr. (**c**) The calculated distance of the penultimate sunrise amplitude fade from the NAA transmitter separated into periods of low (red) and medium (blue) sunspot activity levels. Interference fringe pattern contractions of 4–5 km/yr can be seen.

The analysis of the times of two well defined amplitude fades associated with the passage of the sunrise terminator on the NAA-Halley GCP are summarized in Fig. 3. Panel (a) in Fig. 3 shows the time of the last sunrise amplitude fade on the NAA-Halley path on 28 November for years during the period 1971–2016 where data were available. As we discuss in the methods section, sunrise times were determined for all available data from mid-November to mid-December of each year analysed. A least-squares best fit to the times was made, and the fade time for a representative day was determined, i.e., 28 November. The horizontal blue dashed line indicates the average time of the events, 10:43:30 UT, and the blue dotted lines indicate ±45 s either side of the average. The majority of the fade times lie within the dotted lines, and are consistent with the expectation that the fade occurs as the sunrise terminator passes overhead of the transmitter. Therefore the times remain relatively unchanged throughout the study period, which is an indication that the system timing was reliable. The altitude at which sunrise occurs above the NAA transmitter at 10:43:30 UT is 71.5 ± 1.5 km^32^, where the 1.5 km range is equivalent to the ±45 s identified in panel (a). This altitude is consistent with previous findings^20^, and will be used as the altitude of sunrise along the path for the rest of this study, i.e., sunrise at 71.5 km is important for the phenomenon studied here, not sunrise at ground level. Any long term changes in the altitude of the D-region should be observed as a systematic change in the time of the sunrise fade as the terminator passes overhead of the transmitter. However, the change in the time of sunrise over a small vertical distance range at ~70–75 km is an insensitive test of D-region altitude compared with the expected expansion/contraction in the fringe pattern.

In order to compensate for the change in NAA transmission frequency from 17.8 kHz to 24.0 kHz in 1983, panel (b) in Fig. 3 the time of the penultimate amplitude fade during sunrise has been converted to a time of sunrise at 71.5 km for 24.0 kHz signals. The timing measurements before 1983 typically gave 17.8 kHz fringe distances of ~1600 km at an altitude of 71.5 km, and these have been scaled by the ratio of the two frequencies to give an equivalent fringe distance along the path for 24.0 kHz. A similar calculation has been undertaken for data from 1986, where the only usable data archived during the 1980’s was from amplitude observations of the NSS transmitter (21.4 kHz, Annapolis, Maryland, 38°59′N, 76°27′W). Given the proximity in NAA and NSS transmitter locations, and the similarities in their great circle paths to Halley, the timing of the penultimate amplitude fade of NSS in 1986 was determined and the effective fringe distance from the transmitter scaled up to 24.0 kHz from 21.4 kHz.

Standard deviation errors of the timing measurements of all data point shown in panel 3(b) were calculated, converted into equivalent fringe distance from the transmitter at 71.5 km altitude, and plotted for each year plotted in the panel as a vertical black bar. Typically the standard error in the fringe distance is ~ ±20 km, with a few points having a range of ±50 km. In these latter cases, the large uncertainty comes from either having few points with which to determine the fade time on 28 November, or low timing resolution of the data loggers during that year.

A simple linear least squares fit to all data points in Fig. 3(b) suggests a decrease in the NAA fringe distance from ~2140 km in 1971 to 1980 km in 2016, or a contraction of the fringe pattern of ~3.6 km/yr. Given the sparse data available prior to 1990, and the complicating factor of a different transmission frequency before that as well, a separate linear fit is shown using only the data from 1990 onwards. The shorter period shows a contraction of the fringe pattern by 2.6 km/yr, which is consistent with a weaker shrinking over the last two solar cycles exhibited by LF daytime height trends^18^.

In Fig. 3(b) there is a large scatter about the best fit line, with some suggestion of an 11-year solar cycle influence because of the role of scattered Lyman-α and incident galactic cosmic rays on the nighttime D-region electron number density profiles^24^. This is consistent with potential solar cycle changes in mesopause temperature^33^. Systematic increases in fringe distance are observed during the declining phase of some solar cycles (e.g., 1992–1995, and 2012–2016). This solar cycle influence is investigated in Fig. 3(c) when the data points are separated into periods with low monthly average sunspot number (0–35), medium (36–75) and high (>75) solar activity, as given by the International Space Environmental Services (ISES) sunspot number for November in each year. As there were only three data points in the high solar activity category, spanning only 7 years, those results are not shown. The red data points are associated with low solar activity, while the blue data points are from medium activity periods. Standard errors bars are the same as in panel (b). The panel suggests that there is an outward expansion of the fringe pattern with decreasing solar flux, i.e., from medium levels of sunspot activity to low levels of sunspot activity, consistent with increasing LF phase height during solar declining periods^18^. Least squares fits are shown (red line for low activity, and blue line for medium solar activity). The results show that contractions of the NAA fringe pattern of ~4.7 km/yr occur during low solar activity periods, and ~4.4 km/yr during medium activity periods. The contraction of a mono-chromatic fringe pattern setup between two parallel reflective surfaces (ground and the ionosphere in this case) is consistent with a reduction of the distance between the surfaces^34^. Typically the change in fringe pattern with the quasi-11 year solar cycle is ~100 km, whereas the change over the whole dataset is ~200 km. This contrasts with a LF phase height decrease of ~0.6 km over 50 years, a solar cycle influence of ~1 km, and shorter-term fluctuations (such as the QBO) of ~0.1 km^18^. The VLF transmitter fringe pattern changes studied here are too sparse to resolve fluctuations associated with the QBO, although we note that these are likely to be in the order of 10% of the solar cycle influence^18^, and are therefore ~10 km here, i.e., within the errors bars shown in Fig. 3(a).

### The causes of inference fringe pattern contractions

Over the last 45 years the interference fringe pattern generated by the NAA transmitter has contracted by ~4–5 km/yr over GCP distances of ~2000 km, i.e., a reduction in horizontal scale size of ~10%. Subionospheric radiowave propagation characteristics can be modeled though the use of the Long Wave Propagation Code, LWPC^29^. Typically the wave propagation characteristics are determined by the waveguide boundary conditions consisting of the ground at the lower boundary, and the ionospheric D-region at the upper boundary^19^. Changes in the ground conductivity are not expected, nor investigated here. The D-region is often characterized by electron number density profiles that increase exponentially with altitude, and are described using two parameters – reference height *h*′ (km) and sharpness *β* (km^−1^)^35^. These simplified profiles have been found to adequately represent experimental observations determined from rocket measurements^24^. Nighttime electron number density profiles between 50–110 km were made by calibrated rockets probes launched from Wallops Island (38 °N) in 1964, very relevant to the region studied here^36^. Further nighttime rocket measurements were made at Wallops Island during 1968^37^. Electron number densities at 80, 85, and 90 km were found to be in good agreement with those determined indirectly using VLF subionospheric signals^24^. Nighttime D-region electron number density profiles^24^, described by *h*′ = 85.1 km and *β* = 0.63 km^−1^, also compare well with a semi-empirical statistical D-region model based on rocket-based Faraday rotation data for nighttime conditions^38^ at VLF reflecting electron number densities of <300 el/cm^3^.

Changes in either *h*′ or *β* or both of these parameters can alter the waveguide propagation conditions. LWPC was used to investigate the sensitivity of the NAA nighttime fringe distance to *h*′ and *β*. It was found that the NAA fringe distance was relatively insensitive to *β*, but a contraction of 10% could be achieved through a reduction in nighttime *h*′ by 3 km. Daytime reflection heights at about the same altitude (but higher electron number density) have decreased by 0.6 km over a similar period^18^ which could suggest that some contribution from long-term trends in *β* is necessary to equate the two results. Near the mesopause altitudes a lack of long-term temperature cooling trend has been found^10,15^. However, this behaviour has been attributed to long-term changes in dynamical heating compensating those of CO_2_ cooling, suggesting a sensitive balance between competing influences^16^. In a CO_2_ doubling experiment, northern hemisphere wintertime at about 30° latitude showed dramatic changes of cooling and heating either side of the mesopause^16^ in contrast to other latitudes, but relevant to the study here. Such complex interplay between temperature variations, and dynamical influences on chemical species such as NO and O_2_ (see discussion below) could result in quite different sensitivities to climate change drivers in the daytime or nighttime D-region, as well as subtle altitudinal or latitudinal dependences. Detailed modelling of the sensitivity of the nighttime ionosphere (in the absence of direct daytime solar EUV forcing) to anthropogenic change is required to clarify this point.

Nighttime mid-latitude *h*′ and *β* have been determined previously. Mid-latitude nighttime narrow band radiowave analysis were interpreted from observations made in during 1965–69 ^39^, and suggested *h*′ = 82–87 km, and *β* = *0.5*–*0.8* km^−1^. Nighttime narrow band transmissions during 1995–1997 were also analysed^24^ finding *h*′ = 85.1 ± 0.4 km, and *β* = *0.63* ± *0.04 *km^−1^. Lightning observations were analysed from mid-latitude eastern America in 2004^40^, and estimated *h*′ = 82–85.6 km, and *β* = *0.4*–*0.55 *km^−1^ with an average of 0.45 ± 0.2 km^−1^. Lightning signals were also analysed in 2005 from a similar region^41^, and estimated nighttime *h*′ = 82–87.2 km, but kept *β* constant at 0.65 km^−1^ during the height profile measurement. So, while there is no clear trend with time in the nighttime *h*′ and *β* values previously published, the 3 km reduction in *h*′ reported here is within the ranges historically observed (~5 km). There is some suggestion of a decrease in nighttime *β* with time, where *β* varying over the range 0.65–0.45 km^−1^ could contribute approximately half of the observed horizontal scale contraction. There is also a suggestion to treat *β* as quasi-constant during nighttime^41^ and hence the reduction in horizontal scale size of the NAA interference fringe pattern would primarily be due to the 3 km reduction in *h*′.

The main source of nighttime ionisation in the D-region is through the dissociation of neutral NO by Lyman-α re-radiated by geocoronal neutral hydrogen^42^. Lyman-α is absorbed by O_2_ as it passes through the higher altitude E- and F-regions. Additional ionisation is generated by galactic cosmic rays, although this is less important than Lyman-α at low-mid latitudes^43^. The rapid change of electron density with attitude in the lower D-region is primarily a result of losses attributed to attachment of electrons to O_2_ which increases rapidly with decreasing altitude^43^. Temporal changes in density profiles and reaction rates of these source or loss terms, due to increased infra-red cooling driven by increasing CO_2_ concentrations, is a potential mechanism for the change in *h*′ determined here.

Using subionospheric radiowave observations of the NAA 24.0 kHz transmitter, Cutler, Maine, made from Halley Station, Antarctica, over the period 1971–2016, a ~10% reduction in the scale size of the transmitter nighttime interference fringe pattern has been determined. A novel technique has been developed which is insensitive to any changes in the output power of the transmitter, compensates for the use of different data logging equipment, and can confirm the accuracy of the timing systems in operation over the 45 year study period. Corrections have been made for a change in transmitted frequency from 17.8 kHz to 24.0 kHz in 1978. Subionospheric radiowave propagation modeling suggests that the contraction of the interference fringe pattern about the mid-latitude transmitter is due to a 3 km reduction in the effective height of the ionospheric D-region at about 85 km over 45 years.

## Methods

Data are analysed from a VLF magnetic loop antenna system located at Halley Station, Antarctica (75°36′S, 26°12′W) that has been in operation since 1967. Since then, the amplitude of the NAA VLF transmitter (Cutler, Maine, 44°39′N, 67°17′W) has been logged intermittently using a series of five different instruments. The five instruments that have logged the narrow-band transmissions from NAA are the analogue multi-channel receiver (1971–1975), AVDAS (1981–1990), OMSK (1992–1999), OMNIPAL (2000–2007), and UltraMSK (2012–2017). The instruments have been described in detail^30,31^. During the 1970’s the data were recorded on paper chart and the precise time of radio time signals noted on the paper as a tick mark. Since 1981 the data have been recorded digitally, and the timing synchronized to the Halley station clock. The digital data are simply stored as a time sequence of amplitude measurements, requiring only an identification of the time of the lowest amplitude feature during a fading event. For all years we have determined the times of amplitude fade features for all of the days in mid/late November and early December, where recordings were available. A least squares best fit to the times was made, and a time of the amplitude fade calculated for 28 November in each year. In this way data gaps where either a recording was not made, or the transmitter was off-air on the day, were compensated for. Typically up to twenty days were analysable in each year in order to identify the sunrise timings on the representative day, 28 November, and thus take into account natural ionospheric day-to-day variability. Thus in all ~800 measurements of sunrise fade times were made over the 45 years studied. Variations from the best fit to the times in each year were typically 1–2 minutes normally distributed about the best fit line, and the error bars shown in Fig. 3 are the standard deviations of those residue times.

In some years the NAA transmitter was not recorded at all, and thus no timing estimates could be made. In the 1970’s and 1980’s there were very sparse recordings of NAA made at any time of the year, but the period of November and December provided the most regular sampling of the transmitter, and thus we use the 28 November as the study day. During this period of the year the sunrise terminator passes overhead of the receiver before any other part of the transmitter-receiver GCP, and overhead of the transmitter last. This progression of the terminator from receiver to transmitter means that the modal minima locations are primarily determined by nighttime ionospheric conditions on the remainder of the path from transmitter to minima – resulting in an analysis of the mid-latitude upper atmosphere over ~2000 km between ~44°N and ~25°N (see Fig. 2).

The timings of the amplitude fades observed in the recorded data are converted into distance along the transmitter-receiver GCP using a sunrise almanac. The timing of the final amplitude fade is assumed occur when the terminator is directly overhead of the transmitter^20^. The altitude at which the solar-induced photo-ionisation drives changes in the D-region, and thus influences the received NAA amplitude, can be calculated through the use of the sunrise almanac with appropriate corrections for height and refraction^32^. Uncertainties in the distances determined through converting the calculation of the timing of sunrise were estimated as a standard error of the population of timings that were used to calculate the fade times on 28 November each year. Typically the standard deviation of the mean time of the fades in each year was 1–2 minutes, and the population sample size ranged from 5–20 points, which converts to a distance along the great circle path of ~ ±15–50 km (the terminator sweeps along the Halley to NAA GCP at ~35 km/min in November). The simple linear regression best fit line in Fig. 3(a) showed a gradient of −4.5 km/yr, with a correlation coefficient (r = 0.75 for N = 15) i.e., a significance level of ~99%^44^. By sub-dividing the data values into low and medium sunspot levels, gradients between −4 and −6 km/yr were found, with correlation coefficients of r~0.95, N = 6, indicating significance levels of ~95%.

Data analysed in this study (both the paper records and electronic files) are available at the British Antarctic Survey Polar Data Centre (http://psddb.nerc-bas.ac.uk/data/access/).
